# Screening mammography use and chemotherapy among female stage II colon cancer patients: a retrospective cohort study

**DOI:** 10.1186/1472-6963-10-98

**Published:** 2010-04-19

**Authors:** Xinhua Yu, Alexander M McBean

**Affiliations:** 1 Public Health Program, College of Osteopathic Medicine, Health Professions Division, Nova Southeastern University, Fort Lauderdale, FL, USA; 2 Division of Health Policy and Management, School of Public Health, University of Minnesota, Minneapolis, MN, USA

## Abstract

**Background:**

Although chemotherapy is not a routine recommendation for stage II colon cancer by the U.S. national guidelines, 20-30% of patients have received chemotherapy. This study investigated whether screening mammography use before the cancer diagnosis was associated with chemotherapy use among female elderly patients with stage II colon cancer.

**Methods:**

Retrospective cohort study on 2910 female stage II colon cancer patients aged 67-79 using the Surveillance, Epidemiology and End Results (SEER)-Medicare data (1996-2002). Screening mammography use and chemotherapy use were identified using Medicare claims data. Multivariate logistic regression and Kaplan-Meier curves were used.

**Results:**

About 25% of female elderly patients received chemotherapy. The chemotherapy rates increased from 22% in 1996-1998 to 26% in 2001-2002. After adjusting for socio-demographic variables, tumor characteristics and Charlson index for comorbidities, the odds of receiving chemotherapy were 28% higher among those who had a screening mammogram before the cancer diagnosis than those who did not (OR: 1.28, 95% CI: 1.07-1.54). Those with a prior mammogram also received chemotherapy earlier than those without. In addition, patients with unfavorable tumor characteristics were more likely to receive chemotherapy. Mammography use before the cancer diagnosis was associated with favorable tumor characteristics.

**Conclusions:**

Despite the controversy about the chemotherapy use among stage II colon cancer, female elderly patients still received chemotherapy at a high rate. Our findings suggest that patient's health beliefs and health care seeking behavior, together with physician's recommendation, play important roles in the cancer treatment decision.

## Background

Each year, about 75,000 people are diagnosed with colon cancer in the U.S. [1,2]. Among them, 37% have a stage II cancer diagnosis according to American Joint Committee on Cancer (AJCC) schema, in which cancer is still restricted locally and no positive lymph node is detected. Appropriately treated, the five-year relative survival rate for these patients is about 80% [1].

The primary treatment for stage II colon cancer is surgery. Radiation therapy is not recommended for treating stage II colon cancer because of the side effects on other abdominal organs. Since the 1990s, it has been established that adjuvant chemotherapy can significantly improve survival among patients with stage III cancer (cancer with positive lymph nodes detected) [3]. However, the effectiveness and long term benefits of adjuvant chemotherapy for patients with stage II cancer are still uncertain. The survival benefit might be only 2-5% [4], and the complications of chemotherapy such as severe diarrhea and risk of leucopenia may outweigh the benefits of chemotherapy. Thus, the U.S. national guidelines do not recommend chemotherapy as a routine treatment for those with stage II cancer [3]. Nonetheless, about 20-30% of these patients have received chemotherapy [4].

There are both clinical and non-clinical reasons for patients with stage II colon cancer to receive chemotherapy. Patients with certain tumor characteristics such as a poorly differentiated tumor grade, bowel obstruction or perforation, or tumor extended through serosa (Stage IIb) may have a poor prognosis similar to that of stage III cancer [5]. These patients may be more likely to receive chemotherapy. In addition, when evaluating the risks and benefits of chemotherapy during the treatment decision process, physician's and patient's beliefs in the effect of chemotherapy may play important roles. Some physicians may be more likely to recommend chemotherapy to patients, and a physician's recommendation is the most important determinant for many health care decisions [6]. Furthermore, as suggested by health belief model [7], patient's perception of the vulnerability to colon cancer and its recurrence, the risks and benefits of chemotherapy, and patient's self-efficacy play important roles in forming a patient's own decision. Some patients may be more likely to request chemotherapy or accept the chemotherapy recommendation.

No clinical research has examined the role of patient's health beliefs, revealed in health care seeking behaviors such as screening mammography among women, in the cancer treatment decision. Screening mammography is a highly recommended preventive service by the national guidelines and also well known among women. However, despite the fact that Medicare covers screening mammography since 1994, elderly women who were disabled, with low socio-economic status and insufficient health literacy were less likely to have a screening mammogram within two years. In addition, previous studies have shown that preventive services tend to cluster within certain patients [8]. Regular mammography use has been associated with not only lower breast cancer staging [9] but also higher colorectal cancer screening [10]. Thus, patients with regular mammography use are more health conscious and in general have better health behavior [11,12]. The use of screening mammography before the cancer diagnosis is a good surrogate for a broader health utilization pattern.

In this study, we used Surveillance, Epidemiology and End Results (SEER)-Medicare linked data to compare the chemotherapy use between female stage II colon cancer patients who had a screening mammogram before the cancer diagnosis and those who did not. We hypothesized that those who had a screening mammogram before the cancer diagnosis would also be more likely to receive adjuvant chemotherapy than those who did not have a prior mammogram.

## Methods

### Cohort definition

The 2006 SEER-Medicare linked data were used in this study. The details of SEER-Medicare data can be found at the National Cancer Institute SEER website [13]. Briefly, as of 2002, SEER included all cancer patients residing in 17 geographic areas, covering about 26% of total US populations. For cancer patients 65 years of age and older, 97% of them were linked to Medicare claims data [14]. We identified all incident female colon cancer patients from 1996 to 2002 using the first primary cancer site code (15-23, colon cancer) (n = 49,086) in the SEER Patient Entitlement and Diagnosis Summary File (PEDSF). We excluded patients who had a second malignancy, including colon cancer, within the first year, and included only those with stage II cancer (n = 13,609). Since identifying screening mammography use prior to the cancer diagnosis requires two years of Medicare claims, and mammography rate is significantly lower among people 80 years and older [15], we restricted patients to those with 67 to 79 years of age at the time of cancer diagnosis (n = 5,912). In addition, previous studies have also shown colon cancer patients 80 years and older also have significantly lower chemotherapy rates than younger patients [16]. Similar to other studies using SEER-Medicare data, we hierarchically excluded the cancer patients who did not have both Medicare Part A (hospital insurance) and Part B (medical insurance for office visit) enrollment (Both Part A and B were administered by the US Center for Medicare and Medicaid Service, i.e., CMS) (n = 428), were enrolled in managed care (commercial insurance and health care groups contracted with CMS to manage Medicare beneficiaries) (n = 1,810), or had end-stage renal disease (n = 46) during two years before and one year after the cancer diagnosis. We also excluded those who died (n = 247) or were admitted to hospice (n = 17) within six months around the cancer diagnosis because the treatment decisions for these patients were likely different from others. The date of death was based on Medicare entitlement information included in PEDSF. In-hospital surgical treatment for colon cancer was searched using the International Classification of Diseases, Ninth Revision, Clinical Modification (ICD-9-CM) procedure codes 45.7x, 45.8, 48.4x, 48.5, and 48.6x, in the Medicare Provider Analysis and Review (MedPAR) claims within six months around the cancer diagnosis. Those who did not have a colon cancer related surgery or a surgeon's claim in the Medicare National Claim History (NCH) Carrier during that time window were also excluded (n = 199). Teaching hospital was defined as any hospital with a non-zero indirect cost for medical education in the MedPAR hospitalization claims for the colon surgery.

The study has been approved by the Institutional Review Board (IRB) at the University of Minnesota where the study was initially conducted. The data used in this study were obtained from National Cancer Institute through IMS under the SEER-Medicare data user agreement.

### Adjuvant chemotherapy use

The use of adjuvant chemotherapy treatment was identified from Medicare NCH Carrier claims (The NCH file contain claims for services provided by physicians and stand-alone clinics), Durable Medical Device and Outpatient file claims were searched using Healthcare Common Procedure Coding System (HCPCS) level II codes for commonly used chemotherapy drugs [14]. 99.3% patients who had chemotherapy received 5-fluorouracil (5-FU, J9190) or capecitabine (J8520, J8521), both of which are used either alone, or with leucovorin (J0640), floxuridine (J9200), or oxaliplatin (J9263), irinotecan (J9206). The waiting period for the chemotherapy initiation was defined as the time window from the date of cancer surgery to the date of the first claim containing chemotherapy drugs [17]. Adjuvant chemotherapy is usually started within three months of surgery [16,18]. In our study, 4.2% of patients started chemotherapy after six months. They were excluded (n = 134) because the purpose for the chemotherapy was uncertain [17].

The duration of chemotherapy was defined as the time window from the first to the last claim that contained chemotherapy drug information. Since 1995, typical chemotherapy usually lasts for about six months [3]. We excluded those who were on chemotherapy for more than one year (4.0%, n = 121) because these patients might have a recurrence or the intention of treatment might be a long treatment regimen [19].

### Screening mammography use before the cancer diagnosis

Screening mammography within the two years before the cancer diagnosis was identified using HCPCS codes 76092, G0202, and G0203, and ICD-9-CM code V76.12, in the Medicare NCH Carrier and Outpatient files [15,20].

### Tumor characteristics

Certain tumor characteristics have been related to poor prognosis and may affect chemotherapy decision among stage II colon cancer [5]. We distinguished tumors based on the detailed staging information using SEER extent of diseases codes (defined in SEER EOD-88 3rd edition (1998): 40: invasion through muscularis; 45: extension to adjacent tissue; 50: invasion of/through serosa), and identified bowel obstruction (ICD-9 diagnosis code: 560.89 and 560.90) and bowel perforation (ICD-9 diagnosis codes: 569.83) in the MedPAR claims within four months of the cancer surgery. Those with tumors that have invaded of/through serosa or having bowel obstruction or perforation were Stage IIb colon cancer. Tumor grade from SEER PEDSF was regrouped as well/moderately differentiated and poorly differentiated/other. The "other" group includes unknown grade or ungraded (3.3%, n = 95). They were combined with the poorly differentiated group based on the principle of conservative analysis. In addition, since the number of lymph node examined during the surgery has been related to the quality of surgery and cancer survival, it was grouped as less than 12, and 12 or more [21].

### Visit to oncologists

Chemotherapy is mainly administered by oncologists. In this study, we used all chemotherapy claims from 1996 to 2002 for breast cancer, colorectal cancer, and uterine cancer to identify all physicians who were likely to administer chemotherapy to Medicare cancer patients. Including all these cancer types allows us to have a larger and more complete Medicare claim history to search for possible oncologists and capture those who only saw a small number of elderly patients. An oncologist was defined as a physician who had 5 or more chemotherapy administration or drug claims per year and had seen 2 or more of cancer patients during the study period. Physicians with the HCFA specialty codes (90: medical oncology, 82/83: hematology/oncology, 91: surgical oncology and 70: multiple specialty, 11: internal medicine) accounted for more than 95% of total oncologists identified.

### Patient socio-demographic variables and comorbidities

Patient socio-demographic variables included age group (67-69, 70-74, and 75-79), race/ethnicity (black, white, and other), state buy-in status (state buy-in means the state pays part or all of the patient's Medicare Part B premium or the person is in the Medicaid program), median household income at the census tract based on the 2000 census (If the value was missing, it was imputed from the corresponding zip code median household income), metropolitan residence status (yes versus no), and marital status at the time of cancer diagnosis (married/living with a partner versus living alone). They were obtained from SEER PEDSF.

Patient comorbidities were assessed using Charlson score [22] which was calculated from ICD-9 diagnosis codes in the Medicare NCH, MedPAR and Outpatient claims, in the two years before the month of cancer diagnosis using Deyo and Klabunde modified algorithm [23,24]. The Charlson score was grouped as zero, 1, 2, and 3+. Cancer diagnosis was excluded in calculating the above score.

### Statistical analysis

Descriptive statistics were calculated for patient socio-demographic variables, comorbidities, tumor characteristics, and oncologist visit, stratified by mammography use before the cancer diagnosis. Statistical significance was assessed using t-test for continuous variables and chi-square test for categorical variables. Multivariate adjusted chemotherapy rates were computed as 1/(1+exp(-bx)) from the logistic regression with chemotherapy use (yes/no) as the dependent variable. The time from the cancer surgery to the first chemotherapy use was presented using Kaplan Meier survival curves stratified by prior mammography use. Curves were inverted and also truncated at 160 days to be more interpretable. Odds ratios for the determinants of chemotherapy use were obtained from the above multivariate logistic regression in which all determinants were mutually adjusted. To take account of possible clustering effect of patients, we adopted the Generalized Estimate Equations (GEE) method in the multivariate logistic regression with the hospital where patient received the surgery as the cluster variable. All the statistical analyses were performed using SAS 9.1.4 (Proc GLM, GENMOD, and LIFETEST) (SAS Institute, Cary, NC).

## Results

Our study included 2910 female patients with stage II colon cancer. Their characteristics are shown in Table 1, stratified by screening mammography use status before the cancer diagnosis. Compared with those who did not have a prior mammogram, those who had a prior mammogram were more likely to be younger, white, live in higher income areas, married, healthier, have the colon surgery done in teaching hospitals, and visit an oncologist after the surgery. Further, those who had a prior mammogram had favorable tumor characteristics, were more likely to have 12 or more lymph nodes examined, and were less likely to have bowel obstruction or perforation.

**Table 1:** Patient Characteristics by Screening Mammography Use before the Cancer Diagnosis, SEER-Medicare 1996-2002

		Mammography before cancer diagnosis			
			
		Yes	No	P value	Total
N		1,389	1,521		2,910
					
Age, in years	Mean (SD)	73.8 (3.6)	74.1 (3.6)	0.03	74.0 (3.6)
Age-group	67-69	228 (16.4%)	223 (14.7%)	0.28	451 (15.5%)
	70-74	496 (35.7%)	531 (34.9%)		1027 (35.3%)
	75-79	665 (47.9%)	767 (50.4%)		1432 (49.2%)
					
Race/ethnicity	White	1224 (88.1%)	1286 (84.6%)	0.02	2510 (86.3%)
	Black	95 (6.8%)	133 (8.7%)		228 (7.8%)
	Other	70 (5.0%)	102 (6.7%)		172 (5.9%)
					
State buy-in status	Yes	206 (14.8%)	387 (25.4%)	<0.001	593 (20.0%)
					
Married	Yes	694 (50.0%)	600 (39.5%)	<0.001	1294 (44.5%)
					
Metropolitan residence	Yes	1111 (80.0%)	1259 (82.8%)	0.05	2370 (81.4%)
					
Census tract median household income	<$35,000	551 (39.7%)	669 (44.0%)	0.05	1220 (41.9%)
	$35,000-$60,000	503 (34.3%)	496 (32.6%)		999 (34.3%)
	>$60,000	335 (23.8%)	356 (23.4%)		691 (23.8%)
					
Charlson score	0	396 (28.5%)	539 (35.4%)	<0.001	935 (32.1%)
	1	507 (36.5%)	426 (28.0%)		933 (32.1%)
	2	283 (20.4%)	235 (15.5%)		518 (17.8%)
	3+	203 (14.6%)	321 (21.1%)		524 (18.0%)
					
Teaching hospital		792 (57.9%)	836 (55.0%)	0.26	1628 (56.0%)
					
Tumor grade	Well/moderately differentiated	1051 (75.7%)	1173 (77.1%)	0.36	2224 (76.4%)
					
Extent of disease ^1^	invasion through muscularis	644 (46.4%)	638 (42.0%)	0.03	1282 (44.1%)
	extension to adjacent tissue	550 (39.6%)	621 (40.8%)		1171 (40.2%)
	invasion of/through serosa	79 (5.7%)	95 (6.3%)		174 (6.0%)
	Other	116 (8.4%)	167 (11.0%)		283 (9.7%)
					
Bowel obstruction or perforation		74 (5.3%)	134 (8.8%)	<0.001	208 (7.2%)
					
More than 12 lymph nodes examined		670 (48.2%)	682 (44.8%)	0.07	1352 (46.5%)
					
Oncologist visit		1078 (77.6%)	1148 (75.5%)	0.18	2226 (76.5%)
					
Year	1996-1998	341 (24.6%)	528 (34.7%)	<0.001	869 (29.9%)
	1999-2000	419 (30.2%)	406 (26.7%)		825 (28.4%)
	2001-2002	629 (45.3%)	587 (38.6%)		1216 (41.8%)

Note: 1. The extent of disease for colon cancer is defined in SEER EOD-88 3^rd ^edition (1998) 2. The percentages in the parentheses are column percents

**Figure 1:**
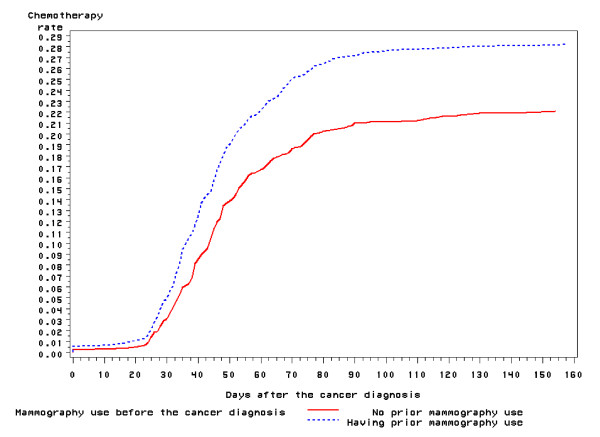
**Time to the Chemotherapy Use among Female Patients with Stage II Colon Cancer, SEER-Medicare 1996-2002**.

Among patients with stage II colon cancer, 25.2% received chemotherapy after the cancer surgery. The crude chemotherapy rates by the mammography use before the cancer diagnosis were presented in Table 2. Those who had a prior mammogram were more likely to receive chemotherapy than those who did not (28.2% versus 22.2%, P < 0.001). Furthermore, younger age, urban residence, better health status, and unfavorable tumor characteristics were related to higher chemotherapy use (Table 2). The time from the cancer surgery to the first chemotherapy use is shown in Figure 1. The chemotherapy was initiated earlier among those who had a prior screening mammogram than those who did not (the median time to the chemotherapy: 52.4 vs. 56.5 days, p = 0.015) (Figure 1). Those who had a prior mammogram also had a higher chemotherapy rate by the fifth month than those who did not (age adjusted chemotherapy rate: 27.4% vs. 21.9%, p = 0.0006).

**Table 2:** Unadjusted Chemotherapy Rate among Female Patients with Stage II Colon Cancer, SEER-Medicare 1996-2002

		Mammography use before cancer diagnosis			
			
		Yes (N = 1,389)	No (N = 1,521)	P value	Total
Total		392 (28.2%)	338 (22.2%)	<0.001	730 (25.1%)
					
Age-group	67-69	78 (34.2%)	65 (29.2%)	<0.001	143 (31.7%)
	70-74	163 (32.7%)	155 (29.2%)		318 (31.0%)
	75-79	151 (22.7%)	118 (15.4%)		269 (18.8%)
					
Race/ethnicity	White	348 (28.4%)	294 (22.9%)	0.28	642 (25.6%)
	Black	24 (25.3%)	24 (18.0%)		48 (21.1%)
	Other	20 (28.6%)	10 (19.6%)		40 (23.3%)
					
State buy-in status	No	350 (29.6%)	285 (25.1%)	<0.001	635 (27.4%)
	Yes	42 (20.4%)	53 (13.7%)		95 (16.0%)
					
Marriage status	Single	169 (24.3%)	164 (17.8%)	<0.001	333 (20.6%)
	Married	223 (32.1%)	174 (29.0%)		397 (30.7%)
					
Metropolitan residence	No	64 (23.0%)	38 (14.5%)	<0.001	102 (18.9%)
	Yes	328 (29.5%)	300 (23.8%)		628 (26.5%)
					
Census tract median household income	<35,000	136 (24.7%)	123 (18.4%)	<0.001	259 (21.2%)
	35,000-60,000	151 (30.0%)	123 (24.8%)		274 (27.4%)
	>60,000	105 (31.3%)	92 (25.8%)		197 (25.1%)
					
Charlson score	0	114 (28.8%)	137 (25.4%)	<0.001	251 (26.8%)
	1	156 (30.8%)	109 (25.6%)		265 (28.4%)
	2	78 (27.6%)	40 (17.0%)		118 (22.8%)
	3+	44 (21.7%)	52 (16.2%)		96 (18.3%)
					
Hospital type	Non-teaching	171 (28.6%)	139 (20.3%)	0.35	310 (24.2%)
	Teaching	221 (27.9%)	199 (23.8%)		420 (25.8%)
					
Tumor grade	Well/moderately differentiated	292 (27.8%)	249 (21.2%)	0.10	541 (24.3%)
	Poorly differentiated/other	100 (29.6%)	89 (25.6%)		189 (27.6%)
					
Extent of diseases	invasion through muscularis	143 (22.2%)	103 (16.1%)	<0.001	246 (19.2%)
	extension to adjacent tissue	173 (31.5%)	148 (23.8%)		321 (27.4%)
	invasion of/through serosa	22 (27.9%)	21 (22.1%)		43 (24.7%)
	Other	54 (46.6%)	66 (39.5%)		120 (42.4%)
					
Bowel obstruction or perforation	No	356 (27.1%)	293 (21.1%)	<0.001	649 (24.0%)
	Yes	36 (48.7%)	45 (33.6%)		81 (38.9%)
					
More than 12 lymph nodes examined	No	187 (26.0%)	180 (21.5%)	0.05	367 (23.6%)
	Yes	205 (30.6%)	158 (23.2%)		363 (26.9%)
					
Oncologist visit	No	20 (6.4%)	15 (4.0%)	<0.001	35 (5.1%)
	Yes	372 (34.5%)	323 (28.1%)		695 (31.2%)
					
Year	1996-1998	82 (24.1%)	112 (21.2%)	0.07	194 (22.3%)
	1999-2000	125 (29.8%)	88 (21.7%)		213 (25.8%)
	2001-2002	185 (29.4%)	138 (23.5%)		323 (26.6%)

Note: The number in each cell is the number of people who received chemotherapy, and the percentage in the parentheses is the corresponding unadjusted chemotherapy rate for that cell

After adjusting for patient socioeconomic variables, comorbidities, tumor characteristics, and oncologist visit, the odds of receiving chemotherapy was 28% higher among those who had a prior mammogram than those who did not (Odds ratio: 1.28, 95% Confidence Interval (CI): 1.07-1.54) (Table 3). As expected, the strongest determinant was whether the patient visited an oncologist after the surgery. Furthermore, patients 75-79 years of age were significantly less likely to receive chemotherapy than the youngest patients (67-69 years of age), while there was no racial difference in the adjusted chemotherapy rates. In addition, the adjusted chemotherapy rates were higher among those who did not have state subsidy for Medicare premium, were married, lived in metropolitan areas and had unfavorable tumor characteristics. Particularly, the adjusted chemotherapy rate was 39.3% for those with bowel obstruction or perforation, and 23.4% for those without (OR: 2.22, 95%CI: (1.55-3.16)). For those with poorly differentiated tumor grade, the adjusted chemotherapy rate was 27.2%, compared with 23.7% for those with well/moderately differentiated tumor grade (p = 0.06). In addition, for those with tumor that only invaded through muscularis, the adjusted chemotherapy rate was only 18.7%, while the adjusted rate was 26.9% and 24.4% for those with tumor that invaded adjacent tissue or through serosa (p < 0.001). Finally, the adjusted chemotherapy rate was also significantly higher in 2001 and 2002 than previous periods (from 21.6% for 1996-1998 to 26.1% for 2001-2002, p for trend = 0.01).

**Table 3:** Determinants of Chemotherapy Use among Female Patients with Stage II Colon Cancer, SEER-Medicare 1996-2002

		Odds ratio	95% CI
Prior mammography use	No	Reference	
	Yes	1.28	(1.07-1.54)
			
Age-group	67-69	Reference	
	70-74	1.00	(0.77-1.30)
	75-79	0.49	(0.39-0.63)
			
Race/ethnicity	White	Reference	
	Black	0.99	(0.67-1.45)
	Other	1.31	(0.84-2.05)
			
State buy-in status	No	Reference	
	Yes	0.56	(0.43-0.73)
			
Marriage status	Single	Reference	
	Married	1.47	(1.20-1.80)
			
Metropolitan residence	No	Reference	
	Yes	1.38	(1.00-1.90)
			
Census tract median household income	<35,000	Reference	
	35,000-60,000	1.14	(0.92-1.42)
	>60,000	0.98	(0.75-1.29)
			
Charlson score	0	Reference	
	1	1.11	(0.87-1.42)
	2	0.86	(0.65-1.14)
	3+	0.77	(0.56-1.04)
			
Hospital type	Non-teaching	Reference	
	Teaching	1.10	(0.89-1.37)
			
Tumor grade	Well/moderately differentiated	reference	
	Poorly differentiated/other	1.14	(0.93-1.39)
			
Extent of diseases	invasion through muscularis	Reference	
	extension to adjacent tissue	1.48	(1.20-1.83)
	invasion of/through serosa	1.27	(0.86-1.89)
	Other	2.91	(2.18-3.90)
			
Bowel obstruction or perforation	No	Reference	
	Yes	2.22	(1.55-3.16)
			
More than 12 lymph nodes examined	No	Reference	
	Yes	1.09	(0.91-1.31)
			
Oncologist visit	No	Reference	
	Yes	8.37	(5.78-12.1)
			
Year	1996-1998	Reference	
	1999-2000	1.28	(0.98-1.66)
	2001-2002	1.36	(1.06-1.73)

Note: The logistic model was mutually adjusted for all these determinants.

## Discussion

In this study the odds of receiving chemotherapy were 28% higher among female stage II colon cancer patients who had a screening mammogram before the cancer diagnosis than those who did not have a prior mammogram. They were also more likely to receive the chemotherapy earlier. In addition, mammography use before the cancer diagnosis was associated with favorable tumor characteristics. Since chemotherapy after the surgery was almost always administered by oncologists, we found that visiting an oncologist after the cancer surgery was the most important determinant of receiving chemotherapy. We repeated all the analyses among those with an oncologist visit and found that our conclusions on the determinants of chemotherapy were not changed (Data not shown).

Recent studies, including both randomized clinical trials [25] and observational studies [4,5], failed to demonstrate a clear benefit of chemotherapy for stage II colon cancer. However, we found that the chemotherapy rate among female patients with stage II colon cancer was about 25%, and the rate increased significantly from 1996 to 2002. The chemotherapy rate was highest in youngest patients (age 67-69), suggesting the chemotherapy rate might be even higher among patients younger than age 67.

In addition, because certain factors such as poor tumor characteristics, bowel obstruction and perforation are related to a poor prognosis similar to that of stage III colon cancer [18], patients with unfavorable clinical indications were significantly more likely to receive chemotherapy than those without these clinical characteristics. Our findings are consistent with those of previous studies [16,17,19]. However, more than 20% of patients with favorable tumor characteristics had also received chemotherapy, suggesting that the chemotherapy decision is not solely based on clinical indications. Characteristics related to both physicians and patients may play important roles in the treatment decision process.

As stated earlier, for many health services, the most important factor is physician's recommendation [6]. National guidelines encourage physicians to discuss the risks and benefits of chemotherapy with patients, and inform patients that chemotherapy for stage II colon cancer is not a routine recommendation [3]. Our study suggests that other factors may be important. The patient's knowledge, attitudes, and beliefs towards colon cancer and chemotherapy may play key roles during the treatment decision making process [7]. Further, women who had regular mammograms were found to be more likely to have colorectal cancer screening [10]. Therefore, although regular mammography may reflect health status, mobility, and access to care, patients with regular mammography might be more aggressive in seeking health care and hold stronger beliefs in maintaining good health than those who did not have regular mammography use [11,12]. Screening mammography before the cancer diagnosis could be considered as a good surrogate for health beliefs regarding cancer care in general. As suggested by the health belief model, those who had screening mammograms might perceive a higher risk of recurrence of colon cancer, and/or larger benefits of chemotherapy than patients without prior screening mammograms. In our study, we found that those with a prior mammogram were more likely to have fewer comorbidities and less advanced diseases, and more likely to visit an oncologist after the surgical treatment. Thus, it could be expected that they would be more willing to adopt chemotherapy.

Consistent with other studies, we confirmed that patient socioeconomic factors are important in cancer treatment decision [26]. Despite the uncertain benefits of chemotherapy for stage II colon cancer, patients who had a state subsidy for their Medicare premium or were in the Medicaid program had significantly lower rates of chemotherapy use, suggesting certain barriers to care existed for these Medicare beneficiaries.

Our study is based on a large and representative population. The high accuracy and representativeness of the SEER cancer registries and comprehensive Medicare claims provided a useful tool to examine the pattern of care among cancer patients. Thus, our findings and conclusions may be generalized to the entire U.S. elderly female population. Furthermore, our study has demonstrated that mammography use before the cancer diagnosis can predict patients' behavior in choosing cancer treatment. We know of no other study that has examined the link between screening mammography use and cancer treatment.

Because this was an observational study based on Medicare claims data, there are some potential weaknesses. We did not have detailed clinical information for each patient except for the Charlson score which was based on the diagnosis codes on Medicare claims. Thus, although we were able to include some tumor characteristics and other clinical indications from the SEER data, we were not able to capture the full spectrum of illness that may have affected the decision to use chemotherapy. Since the decision of chemotherapy use for each patient is made individually, we also did not know how physician and patient communicated about the treatment options. Further research is needed to elucidate the interaction between patient and physician during the treatment decision making process. In addition, we found the chemotherapy was initiated about 4 days earlier among those who had a prior screening mammography than those who did not. Although this may not be clinically significant, it is of importance in the view of health care utilization on average, and confirms that those with regular mammography are more aggressive in seeking health care. Furthermore, we did not directly measure the health beliefs because only administrative data were used. We used screening mammography before the cancer diagnosis as a surrogate for health care seeking behavior. Direct measures of health beliefs and health care seeking behaviors would be desirable in future studies. Finally, given the nature of observational study design, we could not establish any causal relationship between mammography use prior to surgery and the receipt of chemotherapy for stage II colon cancer. Nevertheless, our study provided strong evidence that patient's health care seeking behavior might play an important role in the treatment decision process.

## Conclusion

Elderly women with stage II colon cancer had a high rate of chemotherapy despite the controversy around its use. Those who had a screening mammogram in the two years before the cancer diagnosis were more likely to receive chemotherapy than those who did not have a prior mammogram, suggesting that the patients' knowledge, attitudes and beliefs towards cancer and its treatment, and general health care seeking behaviors is very important in the cancer treatment decision. Physicians need to communicate with patients more effectively so that the decision to adopt chemotherapy is made appropriately based on the national guidelines.

## Competing interests

The authors declare that they have no competing interests.

## Authors' contributions

XY contributes to study design, data analysis, result interpretations, and manuscript drafting. AMM contributes to study design, results interpretations, and manuscript revision. Both authors have read and approved the final manuscript.

## Pre-publication history

The pre-publication history for this paper can be accessed here:

http://www.biomedcentral.com/1472-6963/10/98/prepub
